# Supplementary material for: Factors associated with unmet need for limiting childbirth among women living with HIV in Togo: An averaging approach
Source: PLoS One. 2020 May 20;15(5):e0233136. doi: 10.1371/journal.pone.0233136 (PMC7239477; doi:10.1371/journal.pone.0233136)
Supplement: S1 Data — (PDF) [file pone.0233136.s001.pdf]

QUESTIONNAIRE

Sexualité, contraception, fertilité et qualité de vie chez les personnes vivant avec le VIH au Togo.

Date de l'enquête: |___|___|20____|

Identifiant |__________| *ce numéro est constitué de 3 parties : **1-)** le numéro de la région (voir question 7), **2-)** le numéro de structure de prise en charge selon la liste établie par région et **3-)** le numéro attribué à l'individu suivant l'ordre d'enregistrement.*

SECTION 1: DONNEES SOCIO-DEMOGRAPHIQUES

1. Age : |_____| ans

2. Sexe : |____|
Masculin = 1 ; féminin = 2

Si 1, ne pas administrer les questions de la 28 à la 63.

3. Profession : |___|
Salariée secteur public = 1 ; Salariée secteur privé = 2 ; Salariée secteur informel = 3
Ménagère/sans profession = 4

4. Statut matrimonial :......... |___|
En union = 1 ; pas en union = 2 ; ne sait pas = 99

5. Niveau d'instruction : |___|
Non instruit = 1 ; Primaire = 2 ; secondaire = 3 ; supérieur = 4 ; NSP = 99

6. Lieu de résidence : |___|
Urbain= 1 ; rural = 2

7. Région : |___|
Lomé-commune = 1 ; Maritime = 2 ; Plateaux = 3 ; Centrale = 4 ; Kara = 5 ; Savanes = 6

8. Religion : |___|
Pas religion ou religion traditionnelle = 1 ; Islam = 2 ; Christianisme = 3 ;

SECTION 2: DONNEES CLINIQUES, BIOLOGIQUES ET THERAPEUTIQUES

9. Date de découverte de sa séropositivité |____|____|_______|

10. Statut VIH du partenaire? |___|
VIH positif = 1 ; VIH négatif = 2 ; n'a pas fait le test = 3 ; ne sait pas = 99

11. Comment décrivez-vous vos symptômes actuels VIH? |___|
Aucun =1 ; moyen = 2 ; modéré = 3 ; sévère = 4 ; n'a pas envie de répondre = 99

12. Stade clinique actuel : |___|
Stade I = 1 ; Stade II = 2 ; Stade III = 3 ; Stade IV = 4

13. Etes-vous sous traitement ARV ? |___|
Oui = 1 ; Non =2

14. Si oui, date de début du traitement :
|____|____|_______|

15. Schéma thérapeutique : |___|
1ère ligne = 1 ; 2è ligne = 2 ; 3è ligne = 3

16. Quel est le type d'ARV ? |___|
1ère ligne *: TDF+3TC (ou FTC)+EFV =1 ; TDF+3TC (ou FTC)+NVP = 2 ; AZT+3TC+EFV = 3 ; AZT+3TC +NVP = 4 ;*
2ème ligne *: AZT + 3TC + LPV/r = 5 ; AZT + 3TC + ATV/r = 6 ; TDF + 3TC (ou FTC) + ATV/r = 7; TDF + 3TC (ou FTC) + LPV/r = 8; ABC + 3TC + LPV/r = 9.*
Autres = 14. (à compléter si possible) |____|

17. Le nombre de CD4 au dernier bilan : |______|

18. Charge virale au dernier bilan (si disponible) : |_____| copies/ml.

SECTION 3 : STRUCTURE DE PRISE EN CHARGE

(SVP, adressez-vous au responsable de la structure)

19. Nom de la structure de prise en charge |________________________________|

20. Statut de la structure |____|
Etablissement sanitaire public =1 ; Etablissement sanitaire privé = 2 ; Association = 3 ; Ne sait pas = 4.

21. Type de structure |____|
CMS/USP = 1 ; Hôpital de district = 2 ; CHR = 3 ; CHU = 4 ; Autre =9 ;

Préciser |____________________________|

22. Ressources humaines
a. Nombre de Médecins |____|
b. Nombre d'Assistants Médicaux |____|
c. Nombre d'Infirmiers |____|
d. Nombre de Psychologues |____|

23. Les analyses biologiques réalisées dans la structure ou dans un rayon de 3-5km
Oui = 1 ; Non = 2 ; ne sait pas = 3

a. Hémogramme (NFS)	|____|	b. Créatininémie	|____|
c. Glycémie	|____| ;	d. Transaminases	|____|
e. Sérologie VIH	|____| ;	f. TypagelymphocytaireTCD4/CD8	|____|
g. ARN VIH plasmatique (char.virale)	|____|	h. Cholestérol total	|____|
i. HDL	|____|	j. LDL	|____|
k. Triglycérides	|____|	l. Sérologie de la toxoplasmose	|____|

24. Financement de la structure |____|
Public =1 ; Privé = 2 ; Autres = 3
Si Autres précisez |____|

SECTION 4 : ACTIVITE SEXUELLE ET CONTRACEPTION

25. Êtes-vous sexuellement actif(ve)? |____|
Oui = 1 ; Non = 2 ; ne veut pas répondre = 3
Si Q=2 ou 3, aller à question ***28***

26. A quand remonte votre dernier rapport sexuel? |____|
Une semaine = 1 ; un mois = 2 ; 6 mois = 3 ; ne veut pas répondre = 4

27. Combien de partenaires sexuels avez-vous connu dans les six derniers mois? |____|

Ne pas administrer aux hommes, les questions de la 28 à la 63.

28. Avez-vous déjà entendu parler des méthodes contraceptives ? |____|
Oui = 1 (continuer à la question suivante) ; Non =2 (continue à la section suivante) ; Ne veut pas répondre = 3
29. Si oui où ? |____|
Centre de PEC=1 ; hôpital=2 ; médias = 3 ; amie = 4 ; autre = 5

30. Utilisez-vous la contraception? |____|
Oui = 1 (continuer à la question suivante) ; Non =2 (sauter la question suivante) ; Ne veut pas répondre = 3 (sauter les 2 suivantes questions)

31. Si oui, quel type (choisir une seule réponse)? |____|
Condom = 1; Condom + contraception hormonale (pilule/ implant) = 2; Condom + DIU = 3; Condom + stérilisation= 4 ; Contraception hormonale (pilule/implant) = 5 ; DIU = 6 ; Stérilisation = 7 ; Autres = 8 ; ne veut pas répondre = 9.

31-bis. Pour quelles raisons utilisez-vous la contraception (choisir une seule réponse)? |____|
Eviter une grossesse= 1 ; éviter les IST= 2 ; éviter l'infection à VIH à mon partenaire = 3 ; autre = 4, précisez |____|

32. Si non, pourquoi n'utilisez- vous pas régulièrement de contraception? |____|
Ma propre décision= 1 ; Décision de mon partenaire =2 ; Décision conjointe = 3 ; Mon partenaire a le VIH = 4 ; J'essaie de tomber enceinte = 5 ; Ma charge virale est basse et le risque d'infecter mon partenaire est faible = 6 ; Je ne suis sexuellement active = 7 ; ne veut pas répondre = 8

33. Saviez- vous les endroits où l'on peut se les procurez ?
Oui = 1 (continuer à la question suivante) ; Non =2 (sauterla question suivante) ; Ne veut pas répondre = 3

34. Si oui, citer ces lieux

Centre PF = 1; ATBF= 2 ; hôpital = 3 ; pharmacie = 4; centre de PEC= 5 ; marché = 6 ; ASC =7 ; autre= 8

35. Que pensez-vous de l'utilisation de la contraception chez femme PV/VIH ? |____|

Bonne=1 ; mauvaise= 2 ; ne sais pas = 3
Justifier |____|

SECTION 5 : GROSSESSE

36. êtes-vous ou aviez-vous déjà été enceinte? |____|
Oui, précédemment = 1 ; Oui, je suis actuellement enceinte = 2 ; Non= 3 ; Ne veux pas répondre =4
Si Q = 3 ou 4, continuer à la question **47**

37. avez-vous des enfants ? |____|
Oui = 1 ; Non= 2 ; Ne veux pas répondre = 3
Si Q = 2 ou 3, continuer à la question 43

38. Si oui, combien d'enfants avez-vous? |____|

39. Y en a-t-il un ou plus infecté par le VIH?
Oui = 1 ; Non = 2 ; Ne veux pas répondre = 3.

40.Habitez-vous ensemble avec vos enfants? |____|
Oui = 1 ; Non = 2 ; Ne veux pas répondre = 3

41. Si vous avez été enceinte, dites quand l'avez-vous été pour le dernier enfant? |____|
Avait eu l'enfant avant le diagnostic de VIH = 1 ; Avait eu l'enfant après le diagnostic de VIH = 2 ; Ne veux pas répondre = 3

42. Après avoir été diagnostiquée positive au VIH, aviez-vous : |____|
Un avortement spontané= 1 ; Un avortement provoqué = 2 ; Une naissance d'un mort-né = 3 ; Une (des) naissance(s) vivante(s) = 4 ; Autres = 5; Ne veut pas répondre =6
Si Autre préciser |____|

43. Etiez-vous enceinte au moment où vous étiez diagnostiquée VIH-positive? |____|
Oui = 1 ; Non (continue à la question 47) = 2 ; Ne veut pas répondre (continue à la question 47) = 3.

44. Si oui quel était l'issu de la grossesse?
Naissance(s) vivante(s) = 1 ; Mort-né(s) = 2 ; Avortement spontané = 3 ; Avortement provoqué = 4 ; Ne veut pas répondre = 5

45. Actuellement souhaiteriez-vous tomber enceinte? |____|
Oui = 1, Non = 2 ; J'ai le nombre d'enfants que je veux = 3 ; Ne veut pas répondre = 4

46. Lorsque vous avez été diagnostiquée avec le VIH, cela-t-il changé votre opinion sur le nombre d'enfants vous aviez voulu avoir et à quel moment?
Je voulais avoir des enfants plus tôt = 1 ; Ce n'était pas important pour moi= 2 ; J'avais déjà le nombre d'enfants que je voulais= 3 ; Je ne voulais jamais avoir d'enfants = 4 ; Je voulais avoir des enfants plus tard = 5 ; Le diagnostic a fait que je ne voulais plus avoir d'enfants = 6 ; Autre = 7 ; Ne veut pas répondre = 8.

47. Les bonnes possibilités de traitement du VIH ont-elles influencé votre souhait d'avoir des enfants? |____|
Oui = 1, Non = 2 ; Je ne voulais jamais avoir d'enfants = 3 ; Je ne sais pas = 4 Ne veut pas répondre = 5

SECTION 6 : FERTILITE

48. A quel âge avez-vous eu vos premières menstrues? |____|ans

49. Aviez-vous essayé sans succès de tomber enceinte? |____|
Oui = 1, Non = 2 ; Ne veut pas répondre = 3

50. Actuellement avez-vous essayé de tomber enceinte? |____|
Oui = 1, Non (continue à la question 54) = 2 ; Ne veut pas répondre (continue à la question 54)= 3

51. Pendant combien de temps avez-vous essayé de tomber enceinte? |____|
<6 mois= 1 ; 6–18 mois = 2 ; >18 mois = 3 ; Ne veut pas répondre = 4.

52. Avez-vous déjà été examinée parun médecin pour savoir votre capacité de tomber enceinte? |____|
Oui = 1, Non (continue à la question 56)= 2 ; Ne veut pas répondre (continue à la question 56)= 3

53. Si oui, où l'examen a-t-il eu lieu? |____|
A l'hôpital = 1 ; Chez un généraliste = 2 ; Chez un gynécologue privé= 3 ; À l'étranger = 4 ; Autre = 5, précisez |________________________________| *Ne veut pas répondre = 6.*

54. Durant les 12 derniers mois, aviez-vous des menstruations régulières? |____|

Oui (continue à la SECTION 6) = 1 ; Non = 2 ; Irrégulières mais liées probablement à une cause autre que la ménopause(continue à la SECTION 6) = 3 ; Ne veut pas répondre = 4.

55. Aviez-vous des menstruations régulières durant les 3-12 derniers mois (pas les 2 derniers mois)? |____|
Oui (continue à la question 59)= 1, Non = 2 ; Ne veut pas répondre= 3

56. Aviez-vous vos menstruations durant les 12 derniers mois? |___|
Oui (continue à la question 61) = 1, Non = 2 ; Ne veut pas répondre (continue à la question 61) = 3

57. Pourquoi n'aviez-vous plus vos menstruations? |____|
Survenue naturelle = 1 ; Causé par une ovariectomie et/ou une hystérectomie = 2 ; À la suite d'une chimiothérapie et/ou radiothérapie contre un cancer = 3 ; Traitement hormonal, implant contraceptif ou similaires = 4 ; autre raison= 5 ; précisez |______________________________|
Ne veut pas répondre = 6.

58. Age auquel vos menstruations se sont arrêtées? |____|ans

59. Avez-vous des symptômes de début de la ménopause ? *Oui = 1, Non = 0*

a.Bouffées de chaleur |____| ; ***b. Sueurs nocturnes*** |____|
c. Les troubles du sommeil |____| ***d. Palpitations*** |____|
e. Douleurs thoraciques/poids sur la poitrine |____| ***g. Essoufflement*** |____|
h. Douleurs pendant les rapports sexuels |____| ***k. Nervosité*** |____|
j. Difficultés de concentration |____| ***l. Anxiété/dépression*** |____|
i. Sécheresse des muqueuses du vagin |____| ***m. Courbatures / fatigue*** |____|
n. Maux de tête |____| ***o. Engourdissement*** |____|
q. Douleur dans les articulations |____| ***p. Vertiges*** |____|
r. Augmentation du poids |____| ***s. Perte de contrôle de la vessie*** |____|

60. Avez-vous un traitement hormonal pour les symptômes de la ménopause?

Oui = 1, Non = 2 ; Ne veut pas répondre = 3.

SECTION 7 : TRANSMISSION MERE-ENFANT DU VIH

61. Savez-vous qu'une mère peut transmettre le VIH à son enfant lors de la grossesse et/ou par l'allaitement maternel ? |____|
Oui = 1, Non = 2 ; Ne veut pas répondre = 3

62. Connaissez-vous les mesures permettant de prévenir la transmission du VIH de la mère à l'enfant ? |____|
Oui = 1, Non = 2 ; Ne veut pas répondre = 3

63. Si oui citez-les, *Oui = 1, Non = 0*

- ***a- Charge virale maternelle indétectable (traitement ARV),*** |____|
- ***b- Nouveau-né est mis sous ARV pendant 4 semaines après la naissance*** |____|
- ***c- Pas d'allaitement maternel*** |____|
- ***d- Pratique de la césarienne*** |____|

SECTION 8 : QUALITE DE VIE

Toutes les questions de cette section se rapportent **aux deux dernières semaines.**

64. Domaine I – physique

a. Dans quelle mesure pensez-vous que la douleur (physique) vous empêche defaire ce que vous devez faire? |________|

Pas du tout = 1 ; Un peu = 2 ; Modérément = 3 ; Beaucoup = 4 ; Extrêmement = 5

b. Avez-vous assez d'énergie pour la vie quotidienne? |________|

Pas du tout = 1 ; Un peu = 2 ; Modérément = 3 ; Beaucoup = 4 ; Extrêmement = 5

c. Comment êtes-vous satisfait de votre sommeil? |________|

Très insatisfait = 1 ; Insatisfait = 2 ; Ni insatisfait, ni satisfait = 3 ; Satisfait = 4 ; Très satisfait = 5

d. Comment êtes-vous gêné par des problèmes physiques liés àinfection par le VIH? |________|

Pas du tout = 1 ; Un peu = 2 ; Modérément = 3 ; Beaucoup = 4 ; Extrêmement = 5

65. Domaine II – Psychologique

a. Comment aimez-vous la vie? |________|

Pas du tout = 1 ; Un peu = 2 ; Modérément = 3 ; Beaucoup = 4 ; Extrêmement = 5

b. Comment êtes-vous en mesure de se concentrer? |________|

Pas du tout = 1 ; Un peu = 2 ; Modérément = 3 ; Beaucoup = 4 ; Extrêmement = 5

c. Êtes-vous en mesure d'accepter votre apparence physique? |________|

Pas du tout=1 ; Un peu = 2 ;Modérément = 3 ;Partiellement= 4 ; Complètement = 5

d. Comment êtes-vous satisfait de vous-même? |________|

Très insatisfait = 1 ; Insatisfait = 2 ; Ni insatisfait, ni satisfait = 3 ; Satisfait = 4 ; Très satisfait = 5

e. A quelle fréquence avez-vous des sentiments négatifs tels qu'une mauvaise humeur, dudésespoir, de la dépression, de l'anxiété? |________|

Jamais =1 ; Rarement = 2 ; Souvent = 3 ; Très souvent = 4 ; Toujours = 5

66. Domaine III - Niveau d'indépendance

a. Dans quelle mesure avez-vous besoin d'un traitement médical pour fonctionner dans votre vie quotidienne? |________|

Pas du tout = 1 ; Un peu = 2 ; Modérément = 3 ; Beaucoup = 4 ; Extrêmement = 5

b. Comment êtes-vous en mesure de vous déplacer? |________|

Très mal = 1 ;Mal = 2 ; Ni mal, ni bien = 3 ; Bien = 4 ; Très bien =5.

c. Comment êtes-vous satisfait de votre capacité à effectuer vos activités de la vie quotidienne? |________|

Très insatisfait = 1 ; Insatisfait = 2 ; Ni insatisfait, ni satisfait = 3 ; Satisfait = 4 ; Très satisfait = 5

d. Comment êtes-vous satisfait de votre capacité de travail? |________|

Très insatisfait = 1 ; Insatisfait = 2 ; Ni insatisfait, ni satisfait = 3 ; Satisfait = 4 ; Très satisfait = 5

67. Domaine IV –Les relations sociales

a. Dans quelle mesure estimez-vous être accepté par les gens quevous connaissez? |________|

Pas du tout =1 ; Un peu = 2 ; Modérément = 3 ; Partiellement = 4 ; Complètement = 5

b. Comment êtes-vous satisfait de vos relations personnelles? |________|

Très insatisfait = 1 ; Insatisfait = 2 ; Ni insatisfait, ni satisfait = 3 ; Satisfait = 4 ; Très satisfait = 5

c. Comment êtes-vous satisfait de votre vie sexuelle? |________|

Très insatisfait = 1 ; Insatisfait = 2 ; Ni insatisfait, ni satisfait = 3 ; Satisfait = 4 ; Très satisfait = 5

d. Comment êtes-vous satisfait du soutien que vous recevez de vos amis? |________|

Très insatisfait = 1 ; Insatisfait = 2 ; Ni insatisfait, ni satisfait = 3 ; Satisfait = 4 ; Très satisfait = 5

68. Domaine V – Environnement

a. Comment vous sentez-vous en sécurité dans votre vie quotidienne? |________|

Pas du tout =1 ; Un peu = 2 ; Modérément = 3 ; Partiellement = 4 ; Complètement = 5

b. vivez-vous dans un environnement (physique)sain? |________|

Pas du tout = 1 ; Un peu = 2 ; Modérément = 3 ; Beaucoup = 4 ; Extrêmement = 5

c. Avez-vous assez d'argent pour satisfaire vos besoins? |________|

Pas du tout =1 ; Un peu = 2 ; Modérément = 3 ; Partiellement = 4 ; Complètement = 5

d. L'information dont vous avez besoin dans votre vie au jour le jour est-elledisponible ? |________|

Pas du tout =1 ; Un peu = 2 ; Modérément = 3 ; Partiellement = 4 ; Complètement = 5

e. Avez-vous des opportunités pour les activités deloisirs? |________|

Pas du tout =1 ; Un peu = 2 ; Modérément = 3 ; Partiellement = 4 ; Complètement = 5

f. Comment êtes-vous satisfait des conditions de votre habitation ? |________|

Très insatisfait = 1 ; Insatisfait = 2 ; Ni insatisfait, ni satisfait = 3 ; Satisfait = 4 ; Très satisfait = 5

g. Comment êtes-vous satisfait de votre accès aux services de santé dont vous avez besoins? |________|

Très insatisfait = 1 ; Insatisfait = 2 ; Ni insatisfait, ni satisfait = 3 ; Satisfait = 4 ; Très satisfait = 5

h. Comment êtes-vous satisfait de votre transport? |________|

Très insatisfait = 1 ; Insatisfait = 2 ; Ni insatisfait, ni satisfait = 3 ; Satisfait = 4 ; Très satisfait = 5

69. Domaine VI - Spirituel /Religion / Croyances personnelles

a. Dans quelle mesure vous sentez-vous votre vie a un sens? |________|

Pas du tout =1 ; Un peu = 2 ; Modérément = 3 ; Partiellement = 4 ; Complètement = 5

b. Dans quelle mesure êtes-vous dérangé par des gens qui vous blâment pour votre statut VIH? |________|

Pas du tout =1 ; Un peu = 2 ; Modérément = 3 ; Partiellement = 4 ; Complètement = 5

c. Combien craignez-vous l'avenir? |________|

Pas du tout =1 ; Un peu = 2 ; Modérément = 3 ; Partiellement = 4 ; Complètement = 5

d. Combien vous inquiétez-vous de la mort? |________|

Pas du tout =1 ; Un peu = 2 ; Modérément = 3 ; Partiellement = 4 ; Complètement = 5

70. Qualité de vie, santé générale et perceptions

a. Comment évalueriez-vous votre qualité de vie? |________|

Très mauvaise = 1 ; Mauvaise = 2 ; Ni pauvre, ni bonne = 3 ; Bonne = 4 ; Très bonne = 5

b. Comment êtes-vous satisfait de votre état santé? |________|

Très insatisfait = 1 ; Insatisfait = 2 ; Ni insatisfait, ni satisfait = 3 ; Satisfait = 4 ; Très satisfait = 5
