# Supplementary material for: Factors associated with unmet need for limiting childbirth among women living with HIV in Togo: An averaging approach
Source: PLoS One. 2020 May 20;15(5):e0233136. doi: 10.1371/journal.pone.0233136 (PMC7239477; doi:10.1371/journal.pone.0233136)
Supplement: S2 Data — (PDF) [file pone.0233136.s002.pdf]

QUESTIONNAIRE

Sexuality, contraception, fertility and quality of life in people living with HIV in Togo

---

Date of survey: |___|___|20____|
ID number |_________| *this number is made up of 3 parts: 1-) the number of the region (see question 7), 2-) the number of the care structure according to the list established by region and 3-) the number assigned to the individual following the order of registration.*

SECTION 1: SOCIO-DEMOGRAPHIC DATA

1. Age : |_____| **years**

2. Sexe : |____|
Male = 1 ; female = 2

If 1, do not administer questions from 28 to 63.

3. Profession : |___|
Public sector employee = 1; Private sector employee = 2; Employee in the informal sector = 3
Housewife / without profession = 4

4. Marital status:......... |___|
In union = 1; not in union = 2; don't know = 99

5. Educational level: |___|
Uneducated = 1; Primary = 2; secondary = 3; higher = 4; don't know = 99

6. Place of residence: |___|
Urban= 1 ; rural = 2

7. Region : |___|
Lomé-commune = 1 ; Maritime = 2 ; Plateaux = 3 ; Centrale = 4 ; Kara = 5 ; Savanes = 6

8. Religion : |___|
No religion or traditional religion = 1 ; Islam = 2 ; Christianity = 3 ;

SECTION 2: CLINICAL, BIOLOGICAL AND THERAPEUTIC DATA

9. Date of discovery of his HIV status |____|____|_______|

10. Partner's HIV status?? |___|
HIV positive = 1; HIV negative = 2; have not been tested = 3; don't know = 99

11. How do you describe your current HIV symptoms? |___|
None = 1; medium = 2; moderate = 3; severe = 4; don't want to answer = 99

12. Current clinical stage: |___|
Stage I = 1 ; Stage II = 2 ; Stage III = 3 ; Stage IV = 4

13. Are you on ARV treatment? |___|
Yes = 1; No = 2

14. If yes, start date of treatment: |____|____|_______|

15. Therapeutic regimen: |___|
1st line = 1; 2nd line = 2; 3rd line = 3

16. What is the type of ARV?? |___|

1st line:: *TDF+3TC (ou FTC)+EFV =1 ; TDF+3TC (ou FTC)+NVP = 2 ; AZT+3TC+EFV = 3 ; AZT+3TC +NVP = 4 ;*
2nd line: *AZT + 3TC + LPV/r = 5 ; AZT + 3TC + ATV/r = 6 ; TDF + 3TC (ou FTC) + ATV/r = 7; TDF + 3TC (ou FTC) + LPV/r = 8; ABC + 3TC + LPV/r = 9.*
Others = 14. (to be completed if possible)|________________________________|

17. The number of CD4 at the last check-up: |______|

18. Viral load at last check-up (if available) : |_____| copies/ml.

SECTION 3 : CARE STRUCTURE

(Please contact the person in charge of the structure)

19. Name of the care structure |____________________________|

20. Status of the structure |____|

Public health establishment = 1; Private health facility = 2; Association = 3; Don't know = 4..

21. Type of structure |____|

Primary structure = 1 ; district hospital= 2 ; regional hospital = 3 ; tertiary hospital = 4 ; other =9 ;

Specify |______________________|

22. Human ressources

a. Number of Doctors |____|
b. Number of Medical Assistants |____|
c. Number of Nurses |____|
d. Number of Psychologists |____|

23. Biological analyzes carried out in the structure or within a radius of 3-5 km

Yes = 1; No = 2; don't know = 3

a. Hemogram	|____|	b. Creatinine level	|____|
c. Glycemia	|____| ;	d. Transaminases	|____|
e. HIV serology	|____| ;	f. Lymphocyte typing TCD4 / CD8	|____|
g. Plasma HIV RNA (viral load)	|____|	h. Total cholesterol	|____|
i. HDL	|____|	j. LDL	|____|
k. Triglycerides	|____|	l. Toxoplasmosis serology	|____|

24. Financing of the structure |____|

Public = 1; Private = 2; Others = 3
If others specify |______________________________________|

SECTION 4 : SEXUAL ACTIVITY AND CONTRACEPTION

25. Are you sexually active? |____|

Yes = 1; No = 2; don't want to answer = 3
If Q = 2 or 3, go to question 28

26. When was your last sexual intercourse? |____|

One week = 1; one month = 2; 6 months = 3; don't want to answer = 4

27. How many sexual partners have you known in the past six months? |____|

Do not administer to men, questions from 28 to 63

28. Have you ever heard of contraceptive methods? |____|
Yes = 1 (continue to the next question); No = 2 (continue to next section); Don't want to answer = 3
29. If yes where? |____|
PEC center = 1; hospital = 2; media = 3; friend = 4; other = 5

30. Do you use contraception? |____|
Yes = 1 (continue to the next question); No = 2 (skip the next question); Don't want to answer = 3 (skip the next 2 questions)

31. If yes, what type (choose only one answer)? |____|
Condom = 1; Condom + hormonal contraception (pill / implant) = 2; Condom + IUD = 3; Condom + sterilization = 4; Hormonal contraception (pill / implant) = 5; IUD = 6; Sterilization = 7; Others = 8; don't want to answer = 9.

31-bis. Why do you use contraception (choose only one)? |____|
Avoid pregnancy = 1; avoid STI = 2; avoid HIV infection to my partner = 3; other = 4, specify |____|

32. If not, why don't you use contraception regularly? |____|
My own decision = 1; My partner's decision = 2; Joint decision = 3; My partner has HIV = 4; I am trying to get pregnant = 5; My viral load is low and the risk of infecting my partner is low = 6; I am only sexually active = 7; don't want to answer = 8

33. Did you know where you can get them?
Yes = 1 (continue to the next question); No = 2 (skip the next question); Don't want to answer = 3

34. If yes, name these places

FP center = 1; ATBF = 2; hospital = 3; pharmacy = 4; center of PEC = 5; market = 6; AUC = 7; other = 8

35. What do you think about the use of contraception for women with HIV/AIDS? |____|

Good = 1; bad = 2; don't know = 3
Justify |____|

SECTION 5 : PREGNANCY

36. are you or have you ever been pregnant? |____|
Yes, previously = 1; Yes, I am currently pregnant = 2; No = 3; Don't want to answer = 4
If Q = 3 or 4, continue to question 47

37. do you have children? |____|
Yes = 1; No = 2; Don't want to answer = 3
If Q = 2 or 3, continue to question 43

38. If yes, how many children do you have? |____|

39. Is one or more of you infected with HIV?
Yes = 1; No = 2; Don't want to answer = 3..

40. Do you live together with your children? |____|
Yes = 1; No = 2; Don't want to answer = 3

41. If you were pregnant, say when were you for the last child? |____|
Had the child before diagnosis of HIV = 1; Had child after diagnosis of HIV = 2; Don't want to answer = 3

42. After being diagnosed with HIV, did you: |____|
Spontaneous abortion = 1; Induced abortion = 2; 1 stillborn birth = 3; One (s) live birth (s) = 4; Others = 5; Don't want to answer = 6
If Other specify |____|

43. Were you pregnant when you were diagnosed with HIV-positive? |____|
Yes = 1; No (continue to question 47) = 2; Does not want to answer (continue to question 47) = 3.

44. If yes, what was the outcome of pregnancy?
Live birth (s) = 1; Stillborn (s) = 2; Spontaneous abortion = 3; Induced abortion = 4; Don't want to answer = 5

45. Currently would you like to get pregnant? |____|
Yes = 1, No = 2; I have the number of children I want = 3; Don't want to answer = 4

46. When you were diagnosed with HIV, did it change your opinion of how many children you wanted to have and when?
I wanted to have children earlier = 1; It didn't matter to me = 2; I already had the number of children I wanted = 3; I never wanted to have children = 4; I wanted to have children later = 5; The diagnosis meant that I no longer wanted to have children = 6; Other = 7; Don't want to answer = 8.

47. Have good HIV treatment opportunities influenced your wish to have children?|____|
Yes = 1, No = 2; I never wanted to have children = 3; I don't know = 4 Don't want to answer = 5

SECTION 6 : FERTILITY

48. At what age did you have your first period? |____|**years**

49. Have you tried unsuccessfully to get pregnant? |____|
Yes = 1, No = 2; Don't want to answer = 3

50. Currently have you tried to get pregnant?? |____|
Yes = 1, No (continue to question 54) = 2; Don't want to answer (continue to question 54) = 3

51. How long have you been trying to get pregnant? |____|
<6 months = 1; 6–18 months = 2; > 18 months = 3; Don't want to answer = 4.

52. Have you ever been examined by a doctor to find out if you can get pregnant? |____|
Yes = 1, No (continue to question 56) = 2; Don't want to answer (continue to question 56) = 3

53. If yes, where did the exam take place? |____|
At the hospital = 1; At a general practitioner = 2; At a private gynecologist = 3; Abroad = 4; Other = 5, specify |________________________________| *Don't want to answer = 6.*

54. During the past 12 months, did you have regular periods?? |____|
Yes (continue to SECTION 6) = 1; No = 2; Irregular but probably related to a cause other than menopause (continues in SECTION 6) = 3; Don't want to answer = 4.

55. Did you have regular periods during the last 3-12 months (not the last 2 months)?|____|
Yes (continue to question 59) = 1, No = 2; Don't want to answer = 3

56. Did you have your period during the past 12 months? |____|
Yes (continue to question 61) = 1, No = 2; Don't want to answer (continue to question 61) = 3

57. Why did you no longer have your period? |____|
Natural occurrence = 1; Caused by oophorectomy and / or hysterectomy = 2; Following chemotherapy and / or radiotherapy against cancer = 3; Hormone therapy, contraceptive implant or the like = 4; other reason = 5; specify |_________________________________|

Don't want to answer= 6.

58. Age at which your period stopped? |____|years

59. Do you have symptoms of the onset of menopause? |____|
Yes = 1, No = 0

a. Hot blasts |____| ; ***b. Night sweats*** |____|
c. Sleep disorders |____| ***d. Palpitations*** |____|
e. Chest pain |____| ***g. Shortness of breath*** |____|
h. Pain during intercourse |____| ***k. Nervousness*** |____|
j. Difficulty concentrating |____| ***l. Anxiéty/depression*** |____|
i. Dryness of the mucous membranes of the vagina|____| ***m. Body aches / fatigue*** |____|
n. Headache |____| ***o. Numbness*** |____|
q. Pain in the joints |____| ***p. Vertigo*** |____|
r. Weight gain |____| ***s. Loss of bladder control*** |____|

60. Do you have hormone therapy for menopause symptoms? |____|

Yes = 1, No = 2; Don't want to answer = 3.

SECTION 7 : MOTHER-TO-CHILD TRANSMISSION OF HIV

61. Did you know that a mother can transmit HIV to her child during pregnancy and / or through breastfeeding? |____|
Yes = 1, No = 2; Don't want to answer = 3

62. Do you know the measures to prevent mother-to-child transmission of HIV? |____|
Yes = 1, No = 2; Don't want to answer = 3

63. If yes, quote them, *Yes = 1, No = 0*

- ***a- Undetectable maternal viral load (ARV treatment),*** |____|
- ***b- Newborn baby is put on ARV for 4 weeks after birth*** |____|
- ***c- No breastfeeding*** |____|
- ***d- Practice of cesarean section*** |____|

SECTION 8 : QUALITY OF LIFE

All questions in this section relate to the past two weeks.

64. Domain I – physical

a. How much do you think (physical) pain is preventing you from doing what you need to do? |_______|

Not at all = 1; A little = 2; Moderately = 3; Lots = 4; Extremely = 5

b. Do you have enough energy for everyday life? |_______|

Not at all = 1; A little = 2; Moderately = 3; Lots = 4; Extremely = 5

c. How satisfied are you with your sleep? |_______|

Very dissatisfied = 1; Dissatisfied = 2; Neither dissatisfied nor satisfied = 3; Satisfied = 4; Very satisfied = 5

d. How are you embarrassed by physical problems related to HIV infection? |_______|

Not at all = 1; A little = 2; Moderately = 3; Lots = 4; Extremely = 5

65. Domain II – Psychological

a. How do you like life? |_______|

Not at all = 1; A little = 2; Moderately = 3; Lots = 4; Extremely = 5

b. How are you able to focus? |_______|

Not at all = 1; A little = 2; Moderately = 3; Lots = 4; Extremely = 5

c. Are you able to accept your physical appearance? |_______|

Not at all = 1; A little = 2; Moderately = 3; Partially = 4; Completely = 5

d. How satisfied are you with yourself? |_______|

Very dissatisfied = 1; Dissatisfied = 2; Neither dissatisfied nor satisfied = 3; Satisfied = 4; Very satisfied = 5

e. How often do you have negative feelings such as bad mood, despair, depression, anxiety? |_______|

Never = 1; Rarely = 2; Often = 3; Very often = 4; Always = 5

66. Domain III - Level of independence

a. How much do you need medical treatment to function in your daily life? |_______|

Not at all = 1; A little = 2; Moderately = 3; Lots = 4; Extremely = 5

b. How are you able to get around? |_______|

Very bad = 1; Bad = 2; Neither bad nor good = 3; Good = 4; Very good = 5.

c. How satisfied are you with your ability to perform your daily activities? |_______|

Very dissatisfied = 1; Dissatisfied = 2; Neither dissatisfied nor satisfied = 3; Satisfied = 4; Very satisfied = 5

d. How satisfied are you with your work capacity? |_______|

Very dissatisfied = 1; Dissatisfied = 2; Neither dissatisfied nor satisfied = 3; Satisfied = 4; Very satisfied = 5

67. Domaine IV – Social relations

a. To what extent do you think you are accepted by the people you know? |_______|

Not at all = 1; A little = 2; Moderately = 3; Partially = 4; Completely = 5

b. How satisfied are you with your personal relationships? |_______|

Very dissatisfied = 1; Dissatisfied = 2; Neither dissatisfied nor satisfied = 3; Satisfied = 4; Very satisfied = 5

c. How satisfied are you with your sex life? |_______|

Very dissatisfied = 1; Dissatisfied = 2; Neither dissatisfied nor satisfied = 3; Satisfied = 4; Very satisfied = 5

d. How satisfied are you with the support you receive from your friends? |_______|

Very dissatisfied = 1; Dissatisfied = 2; Neither dissatisfied nor satisfied = 3; Satisfied = 4; Very satisfied = 5

68. Domain V – Environment

a. How do you feel safe in your daily life? |_______|

Not at all = 1; A little = 2; Moderately = 3; Partially = 4; Completely = 5

b. do you live in a healthy (physical) environment? |_______|

Not at all = 1; A little = 2; Moderately = 3; Lots = 4; Extremely = 5

c. Do you have enough money to meet your needs? |_______|

Not at all = 1; A little = 2; Moderately = 3; Partially = 4; Completely = 5

d. Is the information you need in your day-to-day life available? |_______|

Not at all = 1; A little = 2; Moderately = 3; Partially = 4; Completely = 5

e. Do you have opportunities for leisure activities? |_______|

Not at all = 1; A little = 2; Moderately = 3; Partially = 4; Completely = 5

f. How are you satisfied with the conditions of your home? |_______|

Very dissatisfied = 1; Dissatisfied = 2; Neither dissatisfied nor satisfied = 3; Satisfied = 4; Very satisfied = 5

g. How satisfied are you with your access to the health services you need? |_______|

Very dissatisfied = 1; Dissatisfied = 2; Neither dissatisfied nor satisfied = 3; Satisfied = 4; Very satisfied = 5

h. How are you satisfied with your transport? |_______|

Very dissatisfied = 1; Dissatisfied = 2; Neither dissatisfied nor satisfied = 3; Satisfied = 4; Very satisfied = 5

69. Domain VI - Spiritual / Religion / Personal beliefs

a. How meaningful do you feel your life has? |_______|

Not at all = 1; A little = 2; Moderately = 3; Partially = 4; Completely = 5

b. How much are you bothered by people who blame you for your HIV status? |_______|

Not at all = 1; A little = 2; Moderately = 3; Partially = 4; Completely = 5

c. How much do you fear the future? |_______|

Not at all = 1; A little = 2; Moderately = 3; Partially = 4; Completely = 5

d. How much do you worry about death? |_______|

Not at all = 1; A little = 2; Moderately = 3; Partially = 4; Completely = 5

70. Quality of life, general health and perceptions

a. How would you rate your quality of life? |_______|

Very bad = 1; Bad = 2; Neither poor nor good = 3; Good = 4; Very good = 5

b. How satisfied are you with your health? |_______|

Very dissatisfied = 1; Dissatisfied = 2; Neither dissatisfied nor satisfied = 3; Satisfied = 4; Very satisfied = 5
