# Supplementary material for: Factors associated with unmet need for limiting childbirth among women living with HIV in Togo: An averaging approach
Source: PLoS One. 2020 May 20;15(5):e0233136. doi: 10.1371/journal.pone.0233136 (PMC7239477; doi:10.1371/journal.pone.0233136)
Supplement: Attachment — Submitted filename: Response_to_reviewers.docx [file pone.0233136.s004.docx]

**Authors‘ response**

**Here is a point-by-point response to the Editor and the reviewers’ comments and concerns.**

"-In the analysis of unmet need who is the comparator, women with their FP needs met who desire to not have future children? Or are woman who want to conceive in the comparator? This is an important methodological issue, as if women who want to conceive are in the comparison group than there is a lot of heterogeneity in the comparison group (needs met + desire for future children); these groups are very different.

**Answer**: Thank you for this relevant comment. We agree with the reviewer that the comparison group in our study is very heterogeneous. In the comparator group we have women with their FP needs met who desire to not have future children and those who want to conceive."

Copy-editing: The manuscript needs copyediting by native English speaker to address language corrections starting from the abstract then the main paper. See a few a examples (CAPS) in the abstract

**Answer**: Done

Background: With the LARGE access to antiretroviral treatment...….. the subsequent sentence also needs revision

**Answer**: Done

Methods: A cross-sectional study was conducted between June and August 2016, INCLUDING WLHIV of reproductive age (15–49 years), sexually active and followed-up in HIV-care settings in Centrale and Kara regions, in Togo. Data WERE collected on a face-to-face basis - The whole methods section in the abstract needs revision

**Answer**: Done
